# Cardiometabolic Factor-Specific Metabolic Signatures in Metabolic Dysfunction-Associated Steatotic Liver Disease: Insights from Non-Targeted Metabolomics

**DOI:** 10.3390/antiox15080950

**Published:** 2026-07-30

**Authors:** Keigo Misawa, Kouichi Miura, Kenichi Aizawa, Eri Noguchi, Eiko Ueki, Masako Watanabe, Hitoshi Osaka, Tomonori Yano

**Affiliations:** 1 Division of Gastroenterology, Department of Medicine, Jichi Medical University, Shimotsuke 329-0498, Tochigi, Japan; 2 Department of Translational Research, Clinical Research Center, Jichi Medical University Hospital, 3311-1 Yakushiji, Shimotsuke 329-0498, Tochigi, Japan; 3 Clinical Pharmacology Center, Jichi Medical University Hospital, 3311-1 Yakushiji, Shimotsuke 329-0498, Tochigi, Japan; 4 Department of Pediatrics, Jichi Medical University, 3311-1 Yakushiji, Shimotsuke 329-0498, Tochigi, Japan

**Keywords:** metabolic dysfunction associated steatotic liver disease, cardiometabolic factor, metabolites, non-targeted metabolomics analysis

## Abstract

Oxidative stress plays a crucial role in the development of metabolic dysfunction-associated steatotic liver disease (MASLD). Recently, cardiometabolic factors (CMFs) have become essential components of MASLD diagnosis. However, the extent to which individual CMFs contribute to metabolomic alterations in MASLD remains unclear. We aimed to identify associations between CMFs and metabolite levels in patients with MASLD. Serum samples from 31 patients with MASLD and 21 patients without MASLD were subjected to non-targeted metabolomic analysis using liquid chromatography–mass spectrometry. We identified 35 metabolites whose serum levels differed between the non-MASLD and MASLD groups. Of these, 22 metabolites were annotated, including sphingomyelins, phosphatidylcholines, phosphatidylethanolamine, lysophosphatidylcholines, lysophosphatidylethanolamine, plasmalogens, amino acids, a bile acid, and a phenolic compound. Distinct associations were observed between CMFs and metabolite alterations, including obesity and higher glutamic acid levels, diabetes and higher SM(d36:0) and SM(d38:0) levels, and hypertension and lower LPC(18:1) and LPC(18:2) levels. Although several altered metabolites were linked to oxidative stress-related pathways, direct oxidative stress biomarkers were not measured. Thus, the relationship between these metabolite alterations and redox imbalance remains hypothetical and requires further investigation. However, the findings in the present study provide insight into the metabolic mechanisms linking cardiometabolic dysfunction and may facilitate biomarker discovery.

## 1. Introduction

Metabolic dysfunction-associated steatotic liver disease (MASLD), previously termed nonalcoholic fatty liver disease (NAFLD), is the most prevalent chronic liver disease, affecting an estimated 38% of the global population. The number of MASLD patients continues to increase and is projected to exceed 50% of the world population by 2040 [1]. MASLD can progress to liver cirrhosis and hepatocellular carcinoma and is associated with a wide range of extrahepatic comorbidities, including cardiovascular diseases, chronic kidney diseases, and cancers. Although the development of new pharmacological therapies for MASLD is an urgent need, many clinical trials failed to meet a primary efficacy endpoint. One major reason for these failures is the heterogenous nature of MASLD. Therefore, further characterization of MASLD according to the underlying pathogenic mechanism is required. Oxidative stress is considered a central mechanism in the development and progression of MASLD. Excessive production of reactive oxygen species promotes lipid peroxidation, mitochondrial dysfunction, inflammation, and hepatocellular injury, thereby contributing to disease progression. Identification of metabolites associated with oxidative stress and antioxidant defense may therefore provide important insights into the pathophysiology and heterogeneity of MASLD.

The adoption of the MASLD nomenclature in 2023 introduced a pathophysiologically based classification of steatotic liver disease. In this transition, from NAFLD to MASLD, the diagnosis of MASLD requires at least one cardiometabolic factor (CMF) among five CMFs, including obesity, prediabetes and diabetes mellitus (DM), hypertension (HT), hypertriglyceridemia, and low high-density lipoprotein cholesterol (HDL-C) [2]. As a result, substantial new information has emerged regarding the nature of MASLD. For instance, all-cause mortality is significantly higher in patients with MASLD who have four or five CMFs than in those with only one [3]. Among the CMFs, DM and HT in particular are associated with increased all-cause mortality [3]. Until 2023, fibrosis-based assessments were widely used to predict hepatic outcomes [4]. However, data on extrahepatic events according to liver fibrosis stage have been inconsistent [5,6]. Thus, combining CMF-based and fibrosis-based classifications may provide further characterization and the underlying mechanism of MASLD.

Metabolites, including lipids and amino acids, are promising biomarkers for identifying mechanisms and characterization of MASLD. Non-targeted metabolomics is a technique for analyzing the metabolome that aims to comprehensively detect metabolites with molecular weights of approximately 900 Da or less in biological systems, while highlighting those that play the most relevant roles in various processes [7]. Based on high-performance liquid chromatography coupled with high-resolution mass spectrometry (LC-MS), this approach has emerged as a powerful tool for exploring metabolic changes in samples under different conditions, with great potential in fields such as disease diagnosis [8,9,10]. In addition, many reports have focused on metabolites that distinguish NAFLD with advanced fibrosis from simple steatosis [11,12].

Despite the attractiveness of metabolomics for biomarker discovery, data on serum metabolite levels in patients with NAFLD remain inconsistent. For instance, McGlinchey et al. identified metabolites that were unique for steatosis, NASH, and fibrosis in patients with NAFLD [11]. In contrast, Ooi et al. reported that many metabolites increased in steatosis but showed no further changes with progression to NASH [12]. A suspected reason for these discrepancies is the lack of detailed information on CMFs, including concomitant medications. In addition, little is known about the associations between metabolites and liver-related parameters, including transaminases and the grade of steatosis.

In this study, we performed non-targeted metabolomics analysis based on LC-MS using stored serum samples to elucidate differences in metabolites between two patient groups: the MASLD and the non-MASLD groups. Characteristic serum metabolites contributing to differentiation between the two groups were selected using partial least-squares discriminant analysis (PLS-DA). We then discussed the causes of serum metabolic changes associated with the MASLD state, including the influence of CMFs and liver-related parameters.

## 2. Materials and Methods

### 2.1. Patients

MASLD and non-MASLD patients were recruited at Jichi Medical University Hospital. MASLD was diagnosed using published criteria requiring the presence of steatosis and CMF [2]. Briefly, steatosis was diagnosed by imaging examinations, including ultrasonography, computed tomography (CT), or magnetic resonance imaging (MRI); and the presence of hepato-renal contrast on ultrasonography, lower liver density compared with the spleen on CT, and a signal intensity drop in chemical-shift MRI. MASLD patients had at least one CMF. Criteria of CMF-obesity for Asian people, body mass index (BMI) ≥ 23 or waist circumference ≥ 94 cm for men and ≥ 80 cm for women was used in the present study [2]. CMF-DM: fasting serum glucose ≥ 100 mg/dL or HbA1c ≥ 5.7% or use of antidiabetes drugs (Tx-DM). CMF-HT: blood pressure ≥ 130/85 mmHg or use of antihypertensive drugs (Tx-HT). CMF-triglyceride (TG): TG ≥ 150 mg/dL or use of lipid lowering drugs for dyslipidemia (Tx-DL). CMF-HDL-C: HDL-C < 40 mg/dL for men and <50 mg/dL for women, or Tx-DL. Here, each CMF ≥ 5 years was defined as long-time CMF and <5 years as short-time CMF. The non-MASLD patient group included hepatic hemangioma (*n* = 7), chronic hepatitis C with sustained viral response for more than 24 weeks (*n* = 10), and HBs antigen-positive patients with low titer of HBV-DNA, normal transaminase levels, and no nucleotide analog treatment (*n* = 4). These non-MASLD patients did not have steatosis examined by ultrasonography or CT or MRI examination. In addition, there were no significant differences among three groups in BMI, glucose and HbA1c levels, blood pressure, triglyceride, and HDL-C levels. Exclusion criteria encompassed participants who consumed over 30 g of alcohol per day for men or 20 g per day for women, as well as those with other liver diseases. Laboratory data were obtained from electronic medical records. Non-invasive liver fibrosis biomarker FIB-4 index and Mac-2 binding protein glycosylation isomer (M2BPGi) were calculated or measured according to the published formula or method [4]. The degree of steatosis was determined by FibroScan^Ⓡ^ and CAP ≥ 300 dB/m was defined as severe steatosis.

All patients agreed to participate in the present study, and written informed consent was obtained from all patients. The present study was approved by the Institutional Review Board of Jichi Medical University Hospital (permission number A24-023) and conducted in accordance with the Declaration of Helsinki.

### 2.2. Chemicals, Reagents, and Sample Preparation

Methanol, acetonitrile, ultra-pure water, and formic acid of LC/MS grade were used in all experiments. These reagents were purchased from Wako Pure Chemical Industries (Osaka, Japan).

Fasting venous blood samples were taken, and then sera were stored at −80 °C and then thawed immediately prior to use. Fifty microliters of serum were mixed with 200 μL of methanol in a 1.5 mL polypropylene vial. After vigorous shaking, samples were centrifuged (14,000× *g* for 10 min at 4 °C), after which 50 μL of the upper layer was mixed with 200 μL of methanol/ultra-pure water (1:1, *v*/*v*) in a new 1.5 mL polypropylene vial. Samples were again centrifuged after shaking (14,000× *g* for 5 min at 4 °C), and then 200 μL of the upper layer was collected in a 0.3 mL polypropylene vial. Polypropylene vials previously washed with methanol were used for all preparations.

### 2.3. Non-Targeted Metabolomic Analysis Using LC-QTOF-MS

Liquid chromatography quadrupole time-of-flight mass spectrometry (LC-QTOF-MS) analysis was performed on an LCMS9030 system (Shimadzu, Kyoto, Japan). Serum metabolites were separated on a YMC-Accura Triart C18 column (100 mm × 2.1 mm, 1.9 μm; YMC Corporation, Kyoto, Japan). Mobile phases A and B consisted of 0.1% (*v*/*v*) formic acid in ultra-pure water and 0.1% (*v*/*v*) formic acid in acetonitrile/methanol (1:1, *v*/*v*), respectively. The solvent gradient was as follows: 0% B for 1 min followed by a linear gradient to 5% B from 1 to 3 min, to 90% B from 3 to 7 min, and to 100% B from 7 to 11 min, held at 100% B from 11 to 15 min. Subsequently, the mobile phase was returned to initial conditions over 0.5 min and maintained for 4.5 min until the end of the run. The oven temperature was 45 °C, and the flow rate was 0.32 mL/min. The sample volume injected was 2 μL.

MS data were acquired in both positive (pos) and negative (neg) ion modes over the usual range of 70 to 900 *m*/*z* using electrospray ionization. MS parameters were used with default settings as follows: interface voltage of 4.5 kV (pos) and −3.5 kV (neg), interface temperature of 300 °C, nebulization gas flow of 3 L/min, heating gas flow of 10 L/min, drying gas flow of 3 L/min, heat block temperature of 400 °C, and DL temperature of 250 °C. Before sample measurements, *m*/*z* values were calibrated using a calibrant (ESI Tuning Mix for Ion Trap, Sigma-Aldrich Japan, Tokyo, Japan) so that mass accuracy throughout data collection was within 10 ppm. One representative among samples tested was used as a quality control (QC) sample. The QC sample was injected five times in total at the beginning, middle, and end of the batch to check stability of detection amounts during measurements. The relative abundance of each component was evaluated by the peak intensity of the parent ion (MS1) in full scan mode. Additional measurements were also made in data-dependent auto MS/MS mode to obtain fragmentation ions (MS2s) necessary for structure estimation. Fragmentation was performed using argon as a collision gas at a collision energy of 30 eV with a spread of 15 eV. MS2s were generated simultaneously for the top five ions with an *m*/*z* range between 70 and 900, surpassing an intensity threshold of 2000. Data were collected using LabSolutions software (ver.5.118) for LCMS9030 (Shimadzu).

### 2.4. Data Processing and Statistical Analysis

Raw data (the mzML format) were processed using MS-DIAL (ver.5.1.230129) for peak detection, alignment, and integration [13]. We tried to identify components from the exact mass values of elution peaks, with their elution times as complementary information. Searching the database (“ESI(+)-MS/MS from authentic standards” and “ESI(−)-MS/MS from authentic standards” containing information on 16,481 and 9033 unique compounds, respectively), compound names were assigned based on MS1 exact mass values and MS2 spectra. MS-FINDER (ver. 3.56) was also used to assist with assignments of compounds [14]. A private list of priority compounds was used in conjunction with these databases. As necessary, MS1 ions were searched against the HMDB metabolite database (ver. 5.0, accessed on 8 August 2024), MassBank of North America (accessed on 8 August 2024), MetFrag (accessed on 8 August 2024), and the LIPID MAPS lipid database (accessed on 8 August 2024). If MS2 spectra matched one blood metabolite, this compound was annotated as Rank A. If a significant MS2 spectrum could not be obtained, but the MS1 value matched the only metabolite registered in HMDB, it was also annotated as Rank A. If MS1 and/or MS2 spectra matched, but could not be resolved to a single compound due to multiple candidate metabolite hits, a compound that we judged most likely to be a serum metabolite was annotated as Rank B.

**Figure 1:**
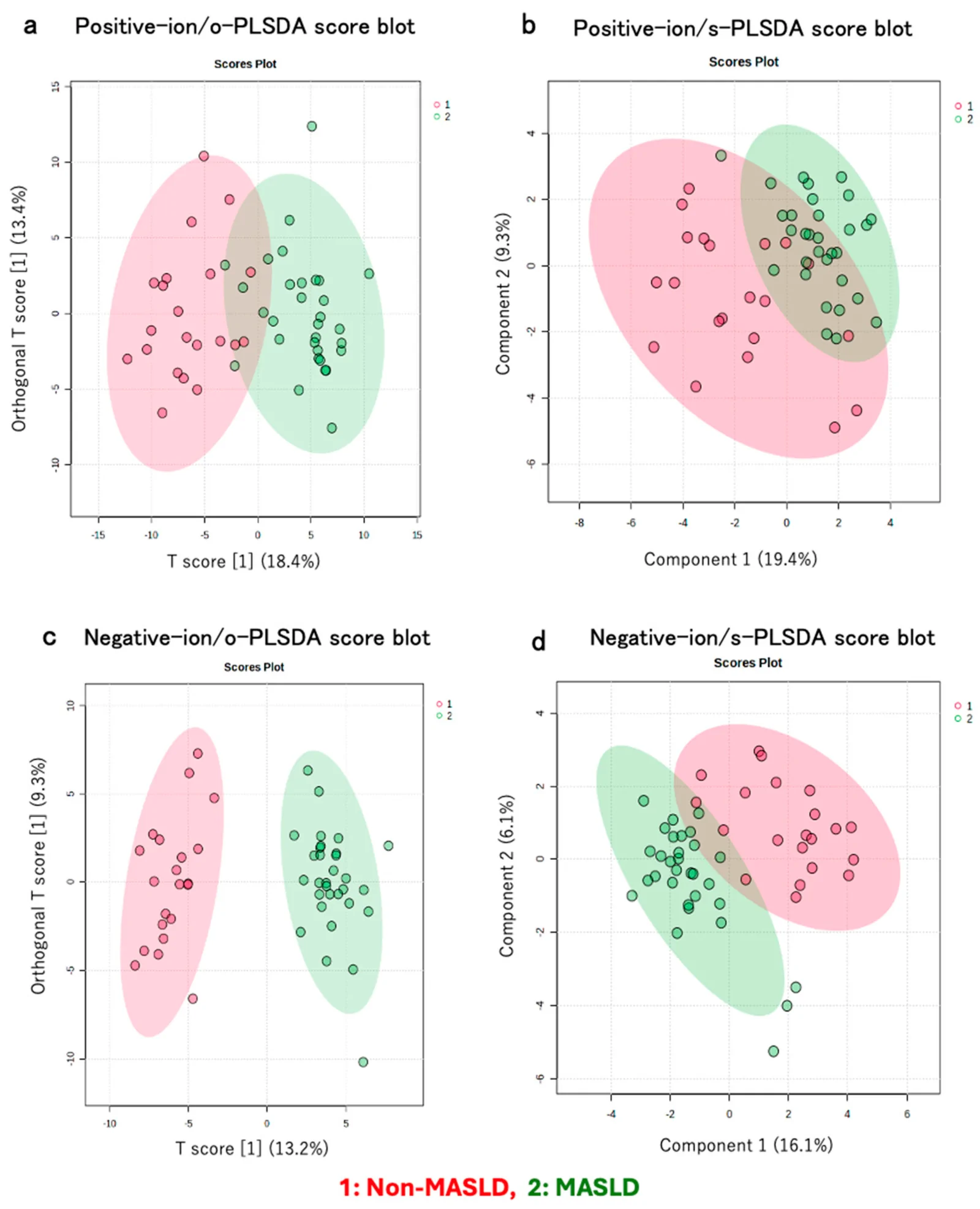
Partial least-squares discriminant analysis (PLS-DA) of serum metabolomic profiles in non-MASLD and MASLD patients. Score plots are shown for positive-ion mode (**a**,**b**) and negative-ion mode (**c**,**d**). Orthogonal PLS-DA (o-PLS-DA) models are presented in (**a**,**c**), whereas supervised PLS-DA (s-PLS-DA) models are shown in (**b**,**d**). Each point represents an individual sample. Clear separation between non-MASLD and MASLD groups was observed in the negative-ion/o-PLS-DA plot (**c**), whereas other plots showed weaker discrimination. Model validation results including R^2^X, R^2^Y, Q^2^, and permutation test analyses for the o-PLS-DA models are shown in Supplementary Figure S1.

Multivariate analyses enabled clear discrimination between the two groups, revealing differences in serum metabolites between them. Sparse PLS-DA (s-PLS-DA) and orthogonal PLS-DA (o-PLS-DA) were performed using MetaboAnalyst version 6.0 (http://www.metaboanalyst.ca, accessed on 1 August 2024). Figure 1 presents score plots generated from positive- and negative-ion datasets using o-PLS-DA and s-PLS-DA models. Candidate metabolites contributing to group discrimination were selected in a data-driven exploratory manner. No predefined VIP threshold was applied. For both o-PLS-DA and s-PLS-DA analyses, metabolites with relatively high variable importance in projection (VIP) values were preferentially selected as candidate discriminatory metabolites. This feature selection procedure should be regarded as exploratory and hypothesis-generating rather than confirmatory. Candidate metabolites obtained from positive- and negative-ion datasets were combined, and duplicated features as well as peaks with poor chromatographic quality were excluded. This procedure yielded 35 metabolites for further evaluation. Non-targeted metabolomics and extraction of characteristic components were performed by referring to previous reports [15,16,17,18]. Model validation of the o-PLS-DA models was performed using R^2^X, R^2^Y, Q^2^, and permutation tests. The validation results are provided in Supplementary Figure S1. Categorical variables were expressed as numbers (percentage), and continuous variables as mean +/− standard deviation. Differences between groups were examined using Pearson’s chi-square test for categorical variables. When data were normally or not normally distributed, differences between groups were assessed using Student’s t-test or the Mann–Whitney U test. Statistical analyses were conducted using STATA version 18 (STATA Corp., Armonk, NY, USA). A value of *p* < 0.05 (*) was considered statistically significant.

### 2.5. Functional Interpretation of Oxidative Stress-Related Metabolites

To discuss the potential relevance of identified metabolites to oxidative stress and antioxidant defense mechanisms, annotated metabolites were interpreted with reference to the published literature and information obtained during metabolite annotation, including the Human Metabolome Database (HMDB) and LIPID MAPS. Particular attention was given to lipid classes previously reported to be associated with redox regulation, lipid peroxidation, membrane protection, and oxidative stress-related metabolic pathways. This interpretation was descriptive in nature and was not used for metabolite selection, classification, or statistical analysis.

## 3. Results

### 3.1. Demographic and Clinical Features of Patients

The characteristics of the patients are shown in Table 1. Compared with non-MASLD group, patients with MASLD had higher BMI, higher transaminase levels, and more severe metabolic impairment, including a higher prevalence of DM and dyslipidemia (DL). Consistently, the proportion of patients receiving any medication for DM or DL was higher in the MASLD group. In line with these findings, the number of cardiometabolic factors (nCMFs) was significantly higher in the MASLD group than in the non-MASLD group. The proportion of patients who fulfill each CMF was also higher among those with MASLD. By contrast, the means of fibrosis markers, FIB4-index, and M2BPGi were similar between the MASLD and non-MASLD groups.

### 3.2. Global Metabolomic Profile Analysis by PLS-DA

Multivariate analysis was performed using PLS-DA to evaluate differences in global serum metabolomic profiles between non-MASLD and MASLD groups. Among the four score plots, the negative-ion/o-PLS-DA plot showed clear separation between the two groups, whereas the other plots demonstrated a weaker degree of discrimination. These findings indicate that MASLD is associated with a distinct serum metabolomic profile (Figure 1).

### 3.3. Serum Metabolite Levels in MASLD Patients

We initially identified 35 metabolites whose serum levels differed between the non-MASLD and MASLD groups. Of those 35 metabolites, 22 metabolites, including 19 Rank A and 3 Rank B metabolites, were successfully identified and selected for further evaluation. Among Rank A and B metabolites, 10 were increased but 11 were decreased in patients with MASLD (Table 2). Metabolites with higher levels in MASLD included three sphingomyelins (SMs), two phosphatidylcholines (PCs), one phosphatidylethanolamine (PE), a bile acid, a phenolic compound, and two amino acids. Although PLS-DA showed that PC(36:4) was a metabolite that discriminates MASLD from non-MASLD, univariate analysis was not significant. Metabolites with lower levels in MASLD included three lysophosphatidylcholines (LPCs), one lysophosphatidylethanolamine (LPE), five plasmalogens, and two PCs.

### 3.4. Metabolite Levels and CMFs

We next compared metabolite levels between MASLD patients with a CMF and those without a CMF (Table 3), because specific CMFs may be associated with the elevation or decline of metabolites. Comparison A was between MASLD patients with the indicated factor and those without that factor; Comparison B was between MASLD and non-MASLD patients, after excluding patients with the indicated factor. Comparison C was between non-MASLD patients with the indicated factor and those without the factor.

MASLD patients with CMF-obesity had higher glutamic acid levels than those without CMF-obesity, and the difference in glutamic acid levels between non-MASLD and MASLD disappeared after excluding patients with CMF-obesity. In addition, these findings were not observed in the non-MASLD group. MASLD patients who met the CMF-DM criteria had higher SM(d36:0) and SM(d38:0) levels than MASLD patients without CMF-DM. The differences in SM(d36:0) and SM(d38:0) levels between non-MASLD and MASLD disappeared after excluding patients with CMF-DM, and these differences were not observed in the non-MASLD group. MASLD patients who met CMF-HT criteria had lower LPC(18:1) and LPC(18:2) levels than those without CMF-HT. The lower LPC(18:1) and LPC(18:2) levels persisted in MASLD patients after excluding those with CMF-HT and were also observed in non-MASLD patients with CMF-HT compared with those without CMF-HT. MASLD patients with CMF-TG had higher PC(36:4) and PC(18:2/18:2) levels than those without CMF-TG. The difference in PC(36:4) between non-MASLD and MASLD patients disappeared after excluding patients with CMF-TG. In contrast, PC(18:2/18:2) levels were lower in MASLD patients than in non-MASLD patients after excluding subjects with CMF-TG.

Duration of CMF may be associated with changes in metabolites. Although we compared each metabolite between patients with long-time and those with short-time CMF, only LPC(18:1) in patients with long time CMF-HT was significantly lower than those with short-time CMF-HT.

Because the number of CMFs (nCMFs) is associated with all-cause mortality [3], we investigated the association between nCMFs and metabolite levels. Only three MASLD patients had two or fewer CMFs in the present study. Therefore, metabolite levels were first compared between patients with nCMFs ≤ 3 (*n* = 10) and those with nCMFs ≥ 4 (*n* = 21), but no metabolites differed significantly between these two groups. Similarly, there were no significant differences in metabolite levels between patients with nCMFs 4 ≤ 4 (*n* = 18) and those with five CMFs (*n* = 13).

### 3.5. Metabolite Levels and Clinical/Laboratory CMF Components

Because CMFs include clinical/laboratory findings and medication, we first investigated the association between metabolite levels and clinical/laboratory components of CMF (Table 4).

MASLD patients with BMI ≥ 23 had higher glutamic acid levels than those without BMI ≥ 23, and the difference in glutamic acid levels between non-MASLD and MASLD disappeared after excluding patients with BMI ≥ 23. These findings were not observed in the non-MASLD group. SM(d36:0) and SM(d38:0) were also higher in MASLD patients with HbA1c ≥ 5.7% compared with those without HbA1c < 5.7. When patients with HbA1c ≥ 5.7% were excluded, the difference in SM(d36:0) and SM(d38:0) levels between non-MASLD and MASLD disappeared, and the differences were not observed in the non-MASLD group. Although LPC(18:2) and LPC(18:1) levels were lower in patients with CMF-HT, these LPC levels did not differ significantly between subjects with blood pressure ≥ 130/85 mmHg and their counterpart. PC(36:4) and PC(18:2/18:2) levels were higher in MASLD with CMF-TG than in those without CMF-TG, and these PCs were higher in MASLD patients with TG ≥ 150 mg/dL than those in those with lower triglyceride levels. When patients with TG ≥ 150 mg/dL were excluded, the difference in PC(36:4) between non-MASLD and MASLD disappeared. In contrast, PC(18:2/18:2) levels were lower in MASLD than in non-MASLD groups after excluding patients with TG ≥ 150 mg/dL (Table 4).

### 3.6. Metabolite Levels and Medications Related to CMFs

We then investigated which classes of drug influenced metabolite levels (Table 5). There were eight MASLD patients with Tx-DM, but no metabolites, including SMs, were significantly altered in MASLD patients with Tx-DM compared with those without.

Although LPC(18:2) and LPC(18:1) were lower in patients with CMF-HT, these LPCs showed no significant differences in comparisons A, B, or C when HT was defined by treatment status. There were no metabolites that were affected by treatment with angiotensin II receptor blockers. PC(36:4) and PC(18:2/18:2) levels were higher in MASLD with CMF-TG than those without CMF-TG, and Tx-DL did not affect the PC(36:4) levels. In contrast, PC(18:2/18:2) levels were lower in MASLD patients with Tx-DL than those without Tx-DL. Furthermore, PC(18:2/18:2) levels were lower in MASLD patients than non-MASLD patients after excluding patients with Tx-DL. Although LPCs and plasmalogens were not associated with CMFs themselves, MASLD patients with Tx-DL had lower LPC(18:1), LPE(18:2), and PC(p16:0/18:2) levels than those without Tx-DL. After excluding patients with Tx-DL, these three metabolites remained lower in MASLD patients than non-MASLD patients. MASLD patients treated with statin had lower LPC(18:2), LPC(18:1), LPE(18:2), PC(p16:0/18:2), and PC(18:2/18:2) levels than those not treated with statins. After excluding patients treated with statin, these five metabolites were still lower in MASLD patients than in non-MASLD patients.

### 3.7. Association Between Metabolite Levels and Liver-Related Biomarkers

We then examined the association between metabolite levels and liver-related biomarkers, including serum transaminase levels and grade of steatosis. Regarding hepatic inflammation, SM(d36:0), SM(d38:0), and phenylalanine levels were higher in the high-ALT group (AST ≥ 40) but not in the high-AST group. SM(d38:0) levels were higher in the severe steatosis group as determined by high CAP values (Table 6).

## 4. Discussion

We identified 22 metabolites that were altered in patients with MASLD, of which 10 metabolites were elevated but 11 were declined. The alterations observed in MASLD patients are consistent with previous reports, including increased SM(d36:0) and SM(d38:0) [19,20], elevated phenylalanyl phenylalanine and glutamic acid [21,22,23], and decreased LPCs [24] and plasmalogens [25]. However, to the best of our knowledge, there were no published data on galloylglycerol and 7-alpha-hydroxy-3-oxo-4-cholestenoic acid in patients with MASLD.

In the present study, SM(d36:0) and SM(d38:0) were increased in MASLD patients with CMF-DM. These findings are in accordance with the previous report that SM(d36:0) levels were higher in patients with prediabetes/diabetes than those without prediabetes/diabetes [19]. Elevation of low-density lipoprotein is a potential mechanism of elevated SMs [26]. We found that poor glycemic control was associated with the elevation of SM(d36:0) and SM(d38:0). Elevation of SM(d36:0) and SM(d38:0) were observed in MASLD patients with HbA1c ≥ 5.7 but not with Tx-DM. After excluding patients with HbA1c ≥ 5.7, the difference in these SMs between non-MASLD and MASLD patients disappeared. Although CMF-DM appeared to contribute to the elevation of SM(d36:0) and SM(d38:0) in MASLD patients, these findings were not observed in non-MASLD patients with CMF-DM, suggesting that additional factors also contribute to the elevation. Jung et al. reported that SM(d36:0) and SM(d38:0) levels correlated with hepatic steatosis and histological inflammation [20]. Consistent with that report, these SMs were elevated in MASLD patients with ALT ≥ 40 in our study. In addition, SM(d38:0) levels were significantly higher in MASLD patients with severe steatosis determined by CAP value ≥ 300. Thus, hepatic inflammation and steatosis may further increase these SMs. Previous reports on blood SM levels in MASLD have been inconsistent, showing either decreases [24] or increases [27]. Another group reported increased SM levels in MASLD with HT but not those with DM [27]. These discrepancies may depend on species of SM. For example, saturated SMs have been reported to be elevated, whereas unsaturated SMs are decreased in MASLD patients [20,24]. The SMs elevated in our study were saturated SMs; therefore, it is important to consider SM species when interpreting these findings.

Two amino acids, phenylalanyl phenylalanine and glutamic acid, were higher in MASLD than non-MASLD patients. Elevated phenylalanine has been associated with ALT concentration [21,28], hepatic steatosis [21,28], and serum triglyceride levels [29], all of which characterized the MASLD patients in our study. We found that MASLD patients with ALT ≥ 40 but not those with ≥ 30 U/L had elevated phenylalanyl phenylalanine levels, suggesting that more severe hepatic inflammation may increase phenylalanine levels. However, there was no significant difference in phenylalanine levels between MASLD patients with CAP value ≥ 300 and those without, using several cut-off values, suggesting that the degree of steatosis does not necessarily increase phenylalanine levels in MASLD. MASLD patients receiving Tx-DL had lower phenylalanine levels than those not receiving Tx-DL, suggesting that lower TG levels induced by Tx-DL may lead to reduced phenylalanine levels. Glutamic acid serves as a key precursor for glutathione synthesis, and alterations in glutathione metabolism have been implicated in oxidative stress and progression of MASLD. Glutamic acid levels were associated with MASLD with obesity, but not with obesity in non-MASLD patients, again suggesting that MASLD-specific factors contribute to glutamic acid elevation. Insulin resistance and liver fibrosis, characteristic features of MASLD, are potential causes of elevated glutamic acid [30]. In addition, increased glutamic acid in MASLD may reflect enhanced degradation of glutathione, which is consumed as an antioxidant in NASH [22].

Galloylglycerol is a plant-derived phenolic metabolite with antioxidative properties. Its elevation may be associated with altered antioxidant responses in MASLD. In the present study, we did not assess vegetable intake, and we did not identify any clinical factors that could explain the elevated galloylglycerol levels. Further studies are therefore required to clarify the mechanisms underlying the increase in galloylglycerol in MASLD.

7-alpha-hydroxy-3-oxo-4-cholestenoic acid is an intermediate metabolite in the synthesis of chenodeoxycholic acid (CDCA), a bile acid, from cholesterol. Serum CDCA levels are higher in patients with NAFLD than those without NAFLD [31], which may partially explain the elevation of 7-alpha-hydroxy-3-oxo-4-cholestenoic acid in MASLD patients.

In contrast to metabolites that elevated in MASLD, several plasmalogen species were decreased in MASLD patients. Plasmalogens are vinyl ether-containing phospholipids that function as endogenous antioxidants and are particularly susceptible to oxidative degradation. Reduced plasmalogen levels have been reported in several conditions associated with increased oxidative stress and lipid peroxidation. Similarly, the reduction in lysophospholipids observed in the present study may be associated with altered phospholipid remodeling under conditions of oxidative stress. Collectively, these findings suggest that the metabolomic alterations identified in MASLD are not only associated with cardiometabolic factors but may also reflect disturbances in oxidative stress-related pathways.

We found that MASLD patients treated with statin had lower levels of LPC(18:1), LPC(18:2), LPE(18:2), PC(p16:0/18:2), and PC(18:2/18:2). These findings are consistent with previous reports that statins reduce certain phospholipids and LPC levels in the circulation [32,33]. LPC is a major component of oxidized LDL [34]. Our previous work showed that oxidized LDL levels were lower in MASLD patients treated with statin than in those not receiving statins [35]. Although we did not measure oxidized LDL levels in the present study, statin therapy may reduce oxidized LDL as well as its component LPCs.

The low levels of lysophospholipids in MASLD patients persisted after excluding patients treated with statin, suggesting that additional factors also influence these metabolite levels. We previously reported that lysophospholipids were increased in the liver of hepatocyte-specific PTEN knockout mice, a model of steatohepatitis, and that remodeling of hepatic phospholipids is a characteristic feature of steatohepatitis [36]. We therefore speculate that incorporation of serum lysophospholipids into hepatic phospholipids may be one mechanism underlying the reduced serum lysophospholipids levels in MASLD. The increased PC(36:4) observed in MASLD may reflect incorporation of these LPCs into PC(36:4).

Although univariate analysis was not significantly different, PLS-DA showed that PC(36:4) was a significant metabolite that discriminates MASLD from non-MASLD. PC(36:4) levels were higher in MASLD patients with CMF-TG than in those without CMF-TG and were also elevated in MASLD patients with TG ≥ 150 mg/dL. PC is essential for the secretion of very low-density lipoprotein, a TG-rich lipoprotein, from hepatocytes into the circulation. After excluding patients with CMF-TG, the difference in PC(36:4) levels between non-MASLD and MASLD patients disappeared, suggesting that CMF-TG is associated with increased PC(36:4) levels in MASLD patients. In contrast, PC(18:2/18:2) levels were higher in MASLD patients with CMF-TG, even though overall PC(18:2/18:2) levels were lower in MASLD than in non-MASLD groups. After excluding patients with CMF-TG, PC(18:2/18:2) levels were lower in MASLD patients than in non-MASLD patients. Because statin use was associated with low PC(18:2/18:2) levels, statin therapy may contribute to reduced PC(18:2/18:2) levels in MASLD patients.

Ferroptosis, an iron-dependent form of cell death driven by lipid peroxidation, is a key feature of MASLD [37]. Susceptibility of ferroptosis is modulated by phospholipid remodeling, including changes in sphingomyelin [38], phosphatidylcholines, phosphatidylethanolamine, and plasmalogens [39]. During ferroptosis, high extracellular concentration of glutamic acid can inhibit the uptake of cystine, which is essential for glutathione synthesis. Furthermore, mitochondria-derived coenzyme Q10 regulates ferroptosis through a glutathione-dependent pathway [40]. Because simple biomarkers for ferroptosis are currently lacking, we are currently investigating whether the specific metabolites identified in the present study could serve as novel ferroptosis biomarkers in MASLD.

The present study demonstrated the alteration of several metabolites that may be linked to oxidative stress and antioxidant defense. However, this study has several limitations. First, it was conducted at a single center and included a relatively small number of participants, and the control group included three different diseases. In addition, we failed to survey dietary intake and physical activities, which may contribute to metabolite changes. As a result, selection bias cannot be excluded. In the present study, five CMFs themselves act as confounding factors (e.g., obesity and diabetes), resulting in very few significant findings when multivariate analysis was performed. Because this is an exploratory study, we conducted two-group comparisons to broadly capture potentially significant findings. Meanwhile, this has a risk of false discovery due to repeated comparisons. In addition, there is a concern regarding a decrease in statistical power in the subgroup analysis due to the small sample size. Although the present study is an exploratory one, metabolite alterations observed in MASLD patients were generally consistent with the previous reports. Firstly, this is, to the best of our knowledge, the first study to describe association between CMFs and metabolite levels in patients with MASLD. Secondly, the present study was a cross-sectional study, and therefore the temporal changes in metabolite profiles during the course of MASLD could not be determined. Thirdly, not all patients underwent liver biopsy despite metabolite levels in patients with liver fibrosis being an interesting research focus. Although we assessed the relationship between metabolites and liver fibrosis using non-invasive biomarkers, no significant findings were obtained. This is partially due to no patients with advanced liver fibrosis in the present study. Fourthly, we did not directly assess oxidative stress-related biomarkers, including glutathione oxidation status, MDA, d-ROMs, 8-OHdG, or oxidized LDL. Therefore, although several altered metabolites have previously been associated with oxidative stress-related pathways, the present study cannot determine whether oxidative stress was actually increased in the study population. Therefore, the proposed relationship between the identified metabolites and oxidative stress should be regarded as descriptive and hypothesis-generating rather than direct evidence of redox imbalance. Future studies combining metabolomic profiling with direct assessment of oxidative stress markers will be necessary to validate these associations.

## 5. Conclusions

In conclusion, we identified distinct associations between cardiometabolic factors and circulating metabolite profiles in patients with MASLD. Diabetes was associated with elevated sphingomyelin levels, obesity with increased glutamic acid, hypertension with reduced lysophosphatidylcholine levels, and weak association between hypertriglyceridemia with increased PC(36:4) levels. These findings suggest that individual cardiometabolic conditions contribute to the heterogeneity of the circulating metabolome in MASLD. Although several altered metabolites were linked to pathways related to oxidative stress and antioxidant defense, direct oxidative stress biomarkers were not assessed. Thus, the relationship between these metabolite alterations and redox imbalance remains hypothetical and requires further validation. Given the exploratory nature of this study, future investigations involving larger multicenter cohorts are warranted to validate these findings and further elucidate the metabolic mechanisms underlying MASLD.

## Figures and Tables

**Table 1:** Characteristics of patients.

Parameter	Non-MASLD	MASLD	*p*
N	21	31	
Male	6 (29%)	10 (32%)	n.s.
Age (y.o.)	59.3 ± 15.6	59.6 ± 15.1	n.s.
BMI	20.9 ± 2.6	27.4 ± 5.4	<0.01
AST (IU/L)	19.7 ± 4.8	43.5 ± 18.4	<0.01
ALT (IU/L)	14.4 ± 4.9	56.7 ± 32.9	<0.01
FBS (mg/dL)	96 ± 11	111 ± 18	<0.01
HbA1c (%)	5.6 ± 0.4	6.3 ± 0.9	<0.01
TC (mg/dL)	208 ± 26.9	196 ± 40.9	n.s.
LDL-C (mg/dL)	119.6 ± 22.7	113.2 ± 35.7	n.s.
HDL-C (mg/dL)	73.4 ± 16.8	57 ± 16.1	<0.01
TG (mg/dL)	78.6 ± 40.6	130.1 ± 71.5	<0.01
Platelet count (×10^4^/μL)	22.5 ± 4.9	21.8 ± 6.4	n.s.
nCMF (mean)	0.9	3.1	<0.01
CMF-obesity	5 (24%)	28 (90%)	<0.01
CMF-DM	4 (19%)	26 (84%)	<0.01
CMF-HT	7 (33%)	27 (87%)	<0.01
CMF-TG	1 (5%)	22(71%)	<0.05
CMF-HDL-C	2 (10%)	17 (55%)	<0.01
Tx-DM	1 (5%)	8 (26%)	<0.05
Tx-HT	5 (24%)	10 (32%)	n.s.
Tx-DL	1 (5%)	15 (48%)	<0.05
FIB-4	1.4 ± 0.9	2.13 ± 1.80	n.s.
M2BPGi (COI)	0.82 ± 0.4	1.46 ± 1.47	n.s.
Current smoking	1 (5%)	1 (3%)	n.s.

BMI, Body mass index; AST, aspartate aminotransferase; ALT, alanine aminotransferase; FBS, fasting blood sugar; HbA1c, hemoglobin A1c; TC, total cholesterol; LDL-C, low-density lipoprotein cholesterol; HDL-C, high-density lipoprotein cholesterol; TG, triglyceride; nCMF, number of cardio metabolic factor; DM, diabetes mellitus; HT, hypertension; Tx, treatment; DL, dyslipidemia; FIB-4, fibrosis-4; M2BPGi, Mac-2 binding protein glycosylation isomer.

**Table 2:** Characteristic metabolites contributing to discrimination between MASLD and non-MASLD groups.

Annotated Compounds	Ion	RT (min)	*m*/*z*	AUC	FC	*p*-Values
SM(d36:0)	negative	14.71	777.617	0.871	1.962	0.00000149
SM(d38:0)	negative	16.08	805.649	0.863	1.795	0.0000108
SM(d40:1)	negative	15.96	831.664	0.816	1.731	0.0000666
PE(18:0/22:6)	negative	13.77	790.544	0.751	1.601	0.0019
Phenylalanyl phenylalanine	positive	6.12	313.156	0.727	1.473	0.00539
PC(18:0/20:5)	negative	13.38	852.581	0.750	1.441	0.00204
Glutamic acid	positive	0.82	148.062	0.814	1.418	0.000142
Galloylglycerol	positive	6.31	245.069	0.719	1.387	0.00728
7-Alpha-hydroxy-3-oxo-4-cholestenoic acid	positive	8.62	431.321	0.767	1.363	0.000934
PC(36:4)	positive	12.61	782.568	0.642	1.349	0.0863
PC(38:3)	positive	14.39	812.625	0.740	1.295	0.00304
ΔLPC(20:0)	negative	10.45	596.396	0.831	0.687	0.0000256
PE(p36:2)	positive	14.89	728.561	0.717	0.687	0.00773
LPC(18:2)	negative	8.57	564.333	0.797	0.676	0.000192
LPC(18:1)	positive	9.11	522.352	0.797	0.676	0.000192
LPE(18:2)	negative	8.7	476.28	0.790	0.676	0.000291
PC(p16:0/18:2)	negative	13.68	786.57	0.833	0.674	0.0000232
ΔPC(p16:0/18:1)	negative	14.48	788.585	0.854	0.671	0.00000533
PC(p34:1)	positive	13.87	744.598	0.737	0.670	0.00347
ΔPC(36:1)	positive	13.89	770.614	0.799	0.659	0.000176
PC(18:2/18:2)	negative	12.75	826.565	0.828	0.644	0.0000312
PC(p36:1)	positive	15.62	772.63	0.825	0.613	0.0000378

The Δ symbol indicates metabolites annotated as Rank B. The others were annotated as Rank A. FC, fold change (MASLD/non-MASLD, mean ratio); RT, retention time; AUC, area under the curve; PC, phosphatidylcholine; LPC, lysophosphatidylcholine; LPE, lysophosphatidylethanolamine; SM, sphingomyelin; SM(d36:0), dihydrosphingomyelin (fatty acid chains 36:0); PE(p36:2), plasmalogen PE (fatty acid chains 36:2). Statistical analysis was performed using the Mann–Whitney U test.

**Table 3:** The association between metabolites and CMFs.

	Annotated Compounds	CMF-Obesity			CMF-DM			CMF-HT			CMF-TG			CMF-HDL-C		
	Comparison	A	B	C	A	B	C	A	B	C	A	B	C	A	B	C
Increased in MASLD	SM(d36:0)				↑	n.s.	n.s.									
	SM(d38:0)				↑	n.s.	n.s.									
	Glutamic acid	↑	n.s.	n.s.												
	PC(36:4)										↑	n.s.	N/A			
Decreased in MASLD	LPC(18:2)							↓	↓	↓						
	LPC(18:1)							↓	↓	↓						
	PC(18:2/18:2)										↑	↓	N/A			

Comparison A shows the difference between MASLD patients with the indicated factor and those without the factor. Comparison B shows the difference between MASLD patients and non-MASLD patients, after excluding patients with the indicated factor. ↑ (increased), ↓ (decreased), and n.s. (no significant difference) in MASLD patients with the indicated factor. Comparison C shows the difference between non-MASLD patients with the indicated factor and those without the factor. ↓ (decreased) and n.s. (no significant difference) in non-MASLD patients with the indicated factor. N/A: Not assessed due to small number of patients. Blanks show no difference in comparisons A, B, and C.

**Table 4:** The association between metabolites and clinical/laboratory findings of CMF.

	Annotated Compounds	BMI ≥ 23 (*n* = 23)			HbA1c ≥ 5.7 (*n* = 25)			BP ≥ 130/85 (*n* = 22)			TG ≥ 150 (*n* = 10)			HDL-C < 40(M), <50 (F) (*n* = 8)		
	Comparison	A	B	C	A	B	C	A	B	C	A	B	C	A	B	C
Increased in MASLD	SM(d36:0)				↑	n.s.	n.s.							↑	↑	N/A
	SM(d38:0)				↑	n.s.	n.s.									
	Phenylalanyl phenylalanine										↑	n.s.	N/A			
	Glutamic acid	↑	n.s.	n.s.							↑	↑	N/A			
	PC(36:4)										↑	n.s.	N/A			
Decreased in MASLD	ΔLPC(20:0)										↑	↓	N/A			
	LPC(18:2)							n.s.	n.s.	n.s.						
	LPC(18:1)							n.s.	n.s.	n.s.						
	PC(18:2/18:2)										↑	↓	N/A			

Comparison A shows the difference between MASLD patients with the indicated factor and those without the factor. Comparison B shows the difference between MASLD patients and non-MASLD patients, after excluding patients with the indicated factor. ↑ (increased), ↓ (decreased), and n.s. (no significant difference) in MASLD patients with the indicated factor. Comparison C shows the difference between non-MASLD patients with the indicated factor and those without the factor. n.s. (no significant difference) in non-MASLD patients with the indicated factor. N/A: Not assessed due to small number of patients. Blanks show no difference in comparisons A, B, and C. BP, blood pressure.

**Table 5:** The association between metabolites and medication related to CMFs.

	Annotated Compounds	SGLT2i (MASLD, *n* = 4) (Non-MASLD, *n* = 0)			DPP4i (MASLD, *n* = 5) (Non-MASLD, *n* = 0)			Tx-HT (MASLD, *n* = 10) (Non-MASLD, *n* = 6)			Calcium-Blocker (MASLD, *n* = 7) (Non-MASLD, *n* = 4)			Tx-DL (MASLD, *n* = 15) (Non-MASLD, *n* = 1)			Statin (MASLD, *n* = 10) (Non-MASLD, *n* = 1)		
	Comparison	A (4 vs. 27)	B (27 vs. 21)	C (0 vs. 21)	A (5 vs. 27)	B (26 vs. 21)	C (0 vs. 21)	A (10 vs. 21)	B (21 vs. 15)	C (21 vs. 15)	A (7 vs. 24)	B (24 vs. 17)	C (4 vs. 17)	A (15 vs. 16	B (16 vs. 20)	C (1 vs. 20)	A (10 vs. 21)	B (21 vs. 20)	C (1 vs. 20)
Increased in MASLD	Phenylalanyl phenylalanine							↓	↑	n.s.				↓	↑	N/A			
	Glutamic acid	↑	↑	N/A															
	PC(38:3)										↑	n.s.	n.s.						
Decreased in MASLD	LPC(18:2)																↓	↓	N/A
	LPC(18:1)													↓	↓	N/A	↓	↓	N/A
	LPE(18:2)													↓	↓	N/A	↓	↓	N/A
	PC(p16:0/18:2)													↓	↓	N/A	↓	↓	N/A
	ΔPC(p16:0/18:1)							↓	↓	n.s.									
	ΔPC(36:1)				↓	↓	N/A												
	PC(18:2/18:2)													↓	↓	N/A	↓	↓	N/A
	PC(p36:1)							↓	↓	n.s.									

Comparison A shows the difference between MASLD patients with the indicated factor and those without the factor. Comparison B shows the difference between MASLD patients and non-MASLD, after excluding patients with the indicated factor. ↑ (increased), ↓ (decreased), and n.s. (no significant difference) in MASLD patients with the indicated factor. Comparison C shows the difference between non-MASLD patients with the indicated factor and those without the factor. n.s. (no significant difference) in non-MASLD patients with the indicated factor. N/A: Not assessed due to small number of patients. Blanks show no difference in comparisons A, B, and C. SGLT2i, sodium glucose co-transporter 2 inhibitor; DPP4i, dipeptidyl peptidase 4 inhibitor.

**Table 6:** The association between metabolite and liver-related markers.

	Annotated Compounds	AST ≥ 40 (*n* = 13)	ALT ≥ 40 (*n* = 21)	CAP ≥ 300 (*n* = 21)
Increased in MASLD	SM(d36:0)		↑	
	SM(d38:0)		↑	↑
	Phenylalanyl phenylalanine		↑	

The difference between MASLD patients with the indicated factor and those without. ↑ (increased) and ↓ (decreased) in MASLD patients with the indicated factor.

## Data Availability

The data that support the findings of the present study are available from the corresponding authors upon reasonable request.

## Supplementary Materials

The following supporting information can be downloaded at: https://www.mdpi.com/article/10.3390/antiox15080950/s1, Figure S1. Validation of orthogonal partial least-squares discriminant analysis (o-PLS-DA) models used for discrimination between non-MASLD and MASLD patients. (A) Positive-ion mode o-PLS-DA model. Model-fit statistics (R^2^X, R^2^Y, and Q^2^) and permutation-test results are shown. R^2^X = 0.195, R^2^Y = 0.650, and Q^2^ = 0.585. Permutation testing yielded R^2^Y = 0.749 (*p* < 0.05) and Q^2^ = 0.578 (*p* < 0.05). (B) Negative-ion mode o-PLS-DA model. Model-fit statistics (R^2^X, R^2^Y, and Q^2^) and permutation-test results are shown. R^2^X = 0.182, R^2^Y = 0.729, and Q^2^ = 0.647. Permutation testing yielded R^2^Y = 0.936 (*p* < 0.05) and Q^2^ = 0.803 (*p* < 0.05).

## Abbreviations

The following abbreviations are used in this manuscript:

BMI	Body mass index
CAP	Controlled attenuation parameter
CMF	Cardiometabolic factor
CT	Computed tomography
DM	Diabetes mellitus
HDL-C	High-density lipoprotein cholesterol
HT	Hypertension
LC-MS	Liquid chromatography coupled with high-resolution mass spectrometry
LPC	Lysophosphatidylcholine
LPE	Lysophosphatidylethanolamine
MRI	Magnetic resonance imaging
M2BPGi	Mac-2 binding protein glycosylation isomer
NAFLD	Nonalcoholic fatty liver disease
nCMFs	Number of cardiometabolic factors
PC	Phosphatidylcholine
PE	Phosphatidylethanolamine
PLS-DA	Partial least-squares discriminant analysis
SM	Sphingomyelin
TG	Triglyceride
Tx-DL	Use of lipid-lowering drugs
Tx-DM	Use of antidiabetic drugs
Tx-HT	Use of antihypertensive drugs

## References

[1] Younossi Z.M., Kalligeros M., Henry L. (2025). Epidemiology of metabolic dysfunction-associated steatotic liver disease. Clin. Mol. Hepatol..

[2] Rinella M.E., Lazarus J.V., Ratziu V., Francque S.M., Sanyal A.J., Kanwal F., Romero D., Abdelmalek M.F., Anstee Q.M., Arab J.P. (2023). A multisociety Delphi consensus statement on new fatty liver disease nomenclature. J. Hepatol..

[3] Li M., Chen W., Deng Y., Xie W. (2024). Impacts of cardiometabolic risk factors and alcohol consumption on all-cause mortality among MASLD and its subgroups. Nutr. Metab. Cardiovasc. Dis..

[4] Miura K., Hayashi H., Kamada Y., Fujii H., Takahashi H., Oeda S., Iwaki M., Kawaguchi T., Tomita E., Yoneda M. (2023). Agile 3+ and Agile 4, noninvasive tests for liver fibrosis, are excellent formulae to predict liver-related events in nonalcoholic fatty liver disease. Hepatol. Res..

[5] Ekstedt M., Hagstrom H., Nasr P., Fredrikson M., Stål P., Kechagias S., Hultcrantz R. (2015). Fibrosis stage is the strongest predictor for disease-specific mortality in NAFLD after up to 33 years of follow-up. Hepatology.

[6] Hagstrom H., Nasr P., Ekstedt M., Hammar U., Stål P., Askling J., Hultcrantz R., Kechagias S. (2019). Cardiovascular risk factors in non-alcoholic fatty liver disease. Liver Int..

[7] Li B., Fu Y., Xi H., Liu S., Zhao W., Li P., Fan W., Wang D., Sun S. (2023). Untargeted Metabolomics Using UHPLC-HRMS Reveals Metabolic Changes of Fresh-Cut Potato during Browning Process. Molecules.

[8] Zang X., Monge M.E., Fernandez F.M. (2019). Mass Spectrometry-Based Non-targeted Metabolic Profiling for Disease Detection: Recent Developments. TrAC Trends Anal. Chem..

[9] Sun Y., Gao H.Y., Fan Z.Y., He Y., Yan Y.-X. (2020). Metabolomics Signatures in Type 2 Diabetes: A Systematic Review and Integrative Analysis. J. Clin. Endocrinol. Metab..

[10] Aderemi A.V., Ayeleso A.O., Oyedapo O.O., Mukwevho E. (2021). Metabolomics: A Scoping Review of Its Role as a Tool for Disease Biomarker Discovery in Selected Non-Communicable Diseases. Metabolites.

[11] McGlinchey A.J., Govaere O., Geng D., Ratziu V., Allison M., Bousier J., Petta S., de Oliviera C., Bugianesi E., Schattenberg J.M. (2022). Metabolic signatures across the full spectrum of non-alcoholic fatty liver disease. JHEP Rep..

[12] Ooi G.J., Meikle P.J., Huynh K., Earnest A., Roberts S.K., Kemp W., Parker B.L., Brown W., Burton P., Watt M.J. (2021). Hepatic lipidomic remodeling in severe obesity manifests with steatosis and does not evolve with non-alcoholic steatohepatitis. J. Hepatol..

[13] Tsugawa H., Cajka T., Kind T., Ma Y., Higgins B., Ikeda K., Kanazawa M., VanderGheynst J., Fiehn O., Arita M. (2015). MS-DIAL: Data-independent MS/MS deconvolution for comprehensive metabolome analysis. Nat. Methods.

[14] Tsugawa H., Kind T., Nakabayashi R., Yukihira D., Tanaka W., Cajka T., Saito K., Fiehn O., Arita M. (2016). Hydrogen Rearrangement Rules: Computational MS/MS Fragmentation and Structure Elucidation Using MS-FINDER Software. Anal. Chem..

[15] Ogiso H., Miura K., Nagai R., Osaka H., Aizawa K. (2024). Non-Targeted Metabolomics Reveal Apomorphine’s Therapeutic Effects and Lysophospholipid Alterations in Steatohepatitis. Antioxidants.

[16] Jacob M., Nimer R.M., Alabdaljabar M.S., Sabi E.M., Al-Ansari M.M., Housien M., Sumaily K.M., Dahabiyeh L.A., Rahman A.M.A. (2022). Metabolomics Profiling of Nephrotic Syndrome towards Biomarker Discovery. Int. J. Mol. Sci..

[17] Saigusa D., Okamura Y., Motoike I.N., Katoh Y., Kurosawa Y., Saijyo R., Koshiba S., Yasuda J., Motohashi H., Sugawara J. (2016). Establishment of Protocols for Global Metabolomics by LC-MS for Biomarker Discovery. PLoS ONE.

[18] Ramabulana A.T., Petras D., Madala N.E., Tugizimana F. (2021). Metabolomics and Molecular Networking to Characterize the Chemical Space of Four Momordica Plant Species. Metabolites.

[19] Im S.S., Park H.Y., Shon J.C., Chung I.-S., Cho H.C., Liu K.-H., Song D.-K. (2019). Plasma sphingomyelins increase in pre-diabetic Korean men with abdominal obesity. PLoS ONE.

[20] Jung Y., Lee M.K., Puri P., Koo B.K., Joo S.K., Jang S.Y., Lee D.H., Jung Y.J., Kim B.G., Lee K.L. (2020). Circulating lipidomic alterations in obese and non-obese subjects with non-alcoholic fatty liver disease. Aliment. Pharmacol. Ther..

[21] Chen Y., Li C., Liu L., Guo F., Li S., Huang L., Sun C., Feng R. (2016). Serum metabonomics of NAFLD plus T2DM based on liquid chromatography-mass spectrometry. Clin. Biochem..

[22] Kalhan S.C., Guo L., Edmison J., Dasarathy S., McCullough A.J., Hanson R.W., Milburn M. (2011). Plasma metabolomic profile in nonalcoholic fatty liver disease. Metabolism.

[23] Chaouche L., Marcotte F., Maltais-Payette I., Tchernof A. (2024). Glutamate and obesity—What is the link?. Curr. Opin. Clin. Nutr. Metab. Care.

[24] Zhou Y., Oresic M., Leivonen M., Gopalacharyulu P., Hyysalo J., Arola J., Verrijken A., Francque S., Van Gaal L., Hyötyläinen T. (2016). Noninvasive Detection of Nonalcoholic Steatohepatitis Using Clinical Markers and Circulating Levels of Lipids and Metabolites. Clin. Gastroenterol. Hepatol..

[25] Puri P., Wiest M.M., Cheung O., Mirshahi F., Sargeant C., Min H., Contos M.J., Sterling R.K., Fuchs M., Zhou H. (2009). The plasma lipidomic signature of nonalcoholic steatohepatitis. Hepatology.

[26] Hammad S.M., Lopes-Virella M.F. (2023). Circulating Sphingolipids in Insulin Resistance, Diabetes and Associated Complications. Int. J. Mol. Sci..

[27] Tiwari-Heckler S., Gan-Schreier H., Stremmel W., Chamulitrat W., Pathil A. (2018). Circulating Phospholipid Patterns in NAFLD Patients Associated with a Combination of Metabolic Risk Factors. Nutrients.

[28] Swierczynski J., Sledzinski T., Slominska E., Smolenski R., Sledzinski Z. (2009). Serum phenylalanine concentration as a marker of liver function in obese patients before and after bariatric surgery. Obes. Surg..

[29] Sun Y., Cai L., Yu B., Zhang H., Zhang Z., Xu X., Yu Y., Li J., Chen C., Xia F. (2025). L-Phenylalanine promotes liver steatosis by inhibiting BNIP3-mediated mitophagy. Mol. Med..

[30] Hasegawa T., Iino C., Endo T., Mikami K., Kimura M., Sawada N., Nakaji S., Fukuda S. (2020). Changed Amino Acids in NAFLD and Liver Fibrosis: A Large Cross-Sectional Study without Influence of Insulin Resistance. Nutrients.

[31] Caussy C., Hsu C., Singh S., Bassirian S., Kolar J., Faulkner C., Sinha N., Bettencourt R., Gara N., Valasek M.A. (2019). Serum bile acid patterns are associated with the presence of NAFLD in twins, and dose-dependent changes with increase in fibrosis stage in patients with biopsy-proven NAFLD. Aliment. Pharmacol. Ther..

[32] Snowden S.G., Grapov D., Settergren M., D’alexandri F.L., Haeggström J.Z., Fiehn O., Hyötyläinen T., Pedersen T.L., Newman J.W., Orešič M. (2014). High-dose simvastatin exhibits enhanced lipid-lowering effects relative to simvastatin/ezetimibe combination therapy. Circ. Cardiovasc. Genet..

[33] Iwase M., Sonoki K., Sasaki N., Ohdo S., Higuchi S., Hattori H., Iida M. (2008). Lysophosphatidylcholine contents in plasma LDL in patients with type 2 diabetes mellitus: Relation with lipoprotein-associated phospholipase A2 and effects of simvastatin treatment. Atherosclerosis.

[34] Liu P., Zhu W., Chen C., Yan B., Zhu L., Chen X., Peng C. (2020). The mechanisms of lysophosphatidylcholine in the development of diseases. Life Sci..

[35] Miura K., Arai N., Goka R., Morimoto N., Watanabe S., Isoda N., Yamamoto H., Kotani K. (2021). Oxidized High-Density Lipoprotein Shows a Stepwise Increase as Fibrosis Progresses in Patients with Nonalcoholic Fatty Liver Disease. Antioxidants.

[36] Tian Y., Jellinek M.J., Mehta K., Seok S.M., Kuo S.H., Lu W., Shi R., Lee R., Lau G.W., Kemper J.K. (2024). Membrane phospholipid remodeling modulates nonalcoholic steatohepatitis progression by regulating mitochondrial homeostasis. Hepatology.

[37] Wang F. (2026). Targeting ferroptosis to halt MASLD and MASH. Trends Endocrinol. Metab..

[38] Lagal D.J., Ortiz-Alcantara A., Pedrajas J.R., McDonagh B., Barcena J.A., Requejo-Aguilar R., Padilla C.A. (2024). Loss of peroxiredoxin 6 alters lipid composition and distribution resulting in increased sensitivity to ferroptosis. Biochem. J..

[39] Shingaki T., Aoki J., Kono N. (2026). Phospholipid remodeling as a key regulator of ferroptosis. J. Lipid Res..

[40] Rochette L., Dogon G., Rigal E., Zeller M., Cottin Y., Vergely C. (2022). Lipid Peroxidation and Iron Metabolism: Two Corner Stones in the Homeostasis Control of Ferroptosis. Int. J. Mol. Sci..

