# Supplementary material for: Cardiometabolic Factor-Specific Metabolic Signatures in Metabolic Dysfunction-Associated Steatotic Liver Disease: Insights from Non-Targeted Metabolomics
Source: Antioxidants (Basel). 2026 Jul 30;15(8):950. doi: 10.3390/antiox15080950 (PMC13510194; doi:10.3390/antiox15080950)
Supplement: Supplementary file 1 [file antioxidants-15-00950-s001.zip › antioxidants-4376154-supplementary.pdf]

(A)

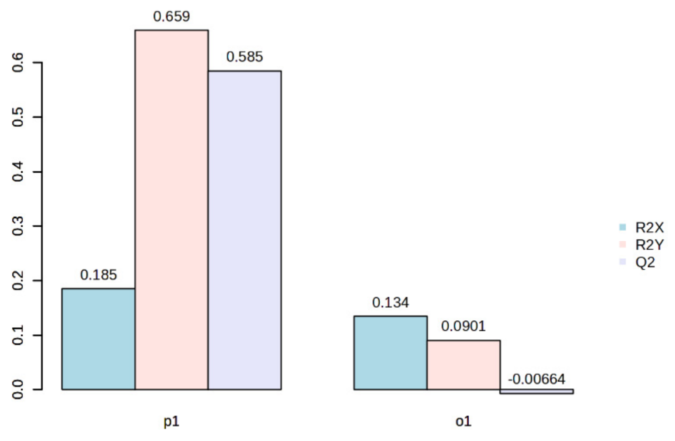

0.659
0.585
0.185
0.134
0.0901
-0.00664
0.0
0.1
0.2
0.3
0.4
0.5
0.6
p1
o1
R2X
R2Y
Q2


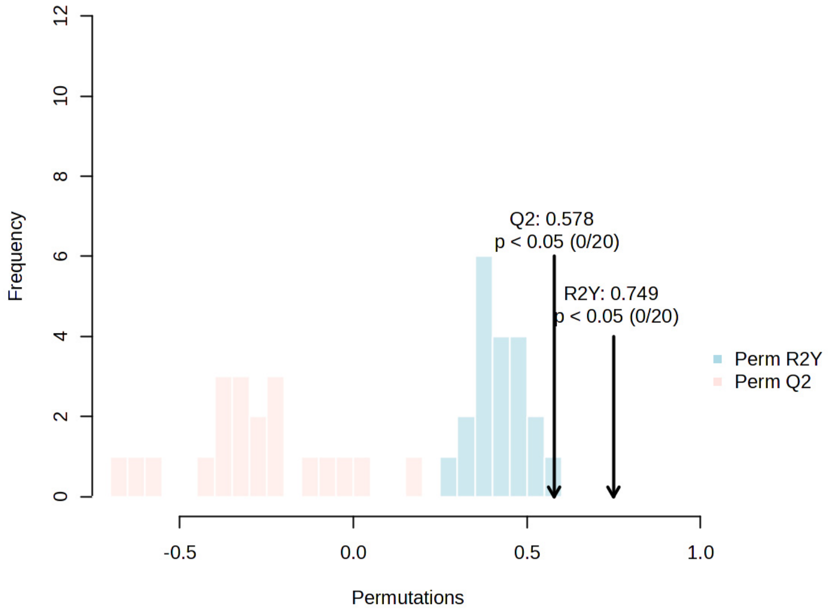

Q2: 0.578
p < 0.05 (0/20)
R2Y: 0.749
p < 0.05 (0/20)
Frequency
0
2
4
6
8
10
12
-0.5
0.0
0.5
1.0
Permutations
Perm R2Y
Perm Q2

(B)

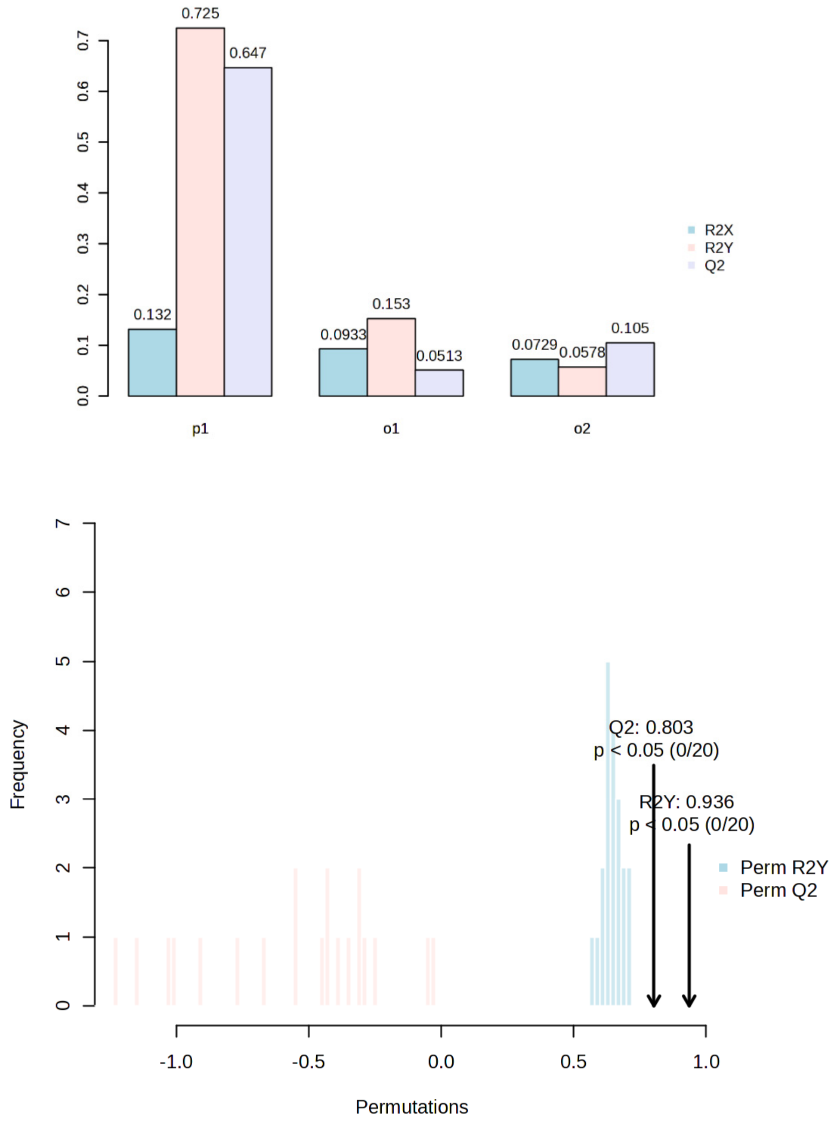


**Supplementary Figure S1.** Validation of orthogonal partial least-squares discriminant analysis (o-PLS-DA) models used for discrimination between non-MASLD and MASLD patients. **(A)** Positive-ion mode o-PLS-DA model. Model-fit statistics ($R^2X$, $R^2Y$, and $Q^2$) and permutation-test results are shown. $R^2X = 0.195$, $R^2Y = 0.650$, and $Q^2 = 0.585$. Permutation testing yielded $R^2Y = 0.749$ ($p < 0.05$) and $Q^2 = 0.578$ ($p < 0.05$). **(B)** Negative-ion mode o-PLS-DA model. Model-fit statistics ($R^2X$, $R^2Y$, and $Q^2$) and permutation-test results are shown. $R^2X = 0.182$, $R^2Y = 0.729$, and $Q^2 = 0.647$. Permutation testing yielded $R^2Y = 0.936$ ($p < 0.05$) and $Q^2 = 0.803$ ($p < 0.05$).
